# Supplementary material for: Characterization and Valorization of the Agricultural Waste Obtained from Lavandula Steam Distillation for Its Reuse in the Food and Pharmaceutical Fields
Source: Molecules. 2022 Feb 28;27(5):1613. doi: 10.3390/molecules27051613 (PMC8911589; doi:10.3390/molecules27051613)
Supplement: Supplementary file 1 [file molecules-27-01613-s001.zip › molecules-1591485-supplementary.pdf]

METHODS

Hydrosol extraction

The components of hydrosols were extracted to characterize the chemical composition as described by Truzzi et al. [1]. Briefly, 10 g of hydrosol were extracted at first with EtOAc (3 x 5 mL); subsequently with Hex (3 x 5 mL), and the combined organic phases were washed with brine (NaCl saturated solution). The organic phase was dried over Na_2SO_4 and concentrated at room temperature under vacuum. The residue was weighted, solubilized in Hex, and analyzed by GC. The extraction was performed in triplicate.

Analysis of the EOs

GC-MS analysis

Analyses were performed on a 7890A gas chromatograph coupled with a 5975C network mass spectrometer (GC-MS) (Agilent Technologies, Milan, Italy). Compounds were separated on an Agilent Technologies HP-5 MS cross-linked poly-5% diphenyl–95% dimethyl polysiloxane (30 m × 0.25 mm i.d., 0.25 μm film thickness) capillary column. The column temperature was initially set at 45 °C, then increased at a rate of 2 °C/min up to 100 °C, then raised to 250 °C at a rate of 5 °C/min and finally held for 5 min. The injection volume was 0.1 μL, with a split ratio 1:20. Helium was used as the carrier gas, at a flow rate of 0.7 mL/min. The injector, transfer line, and ion-source temperatures were 250, 280, and 230 °C, respectively. MS detection was performed with electron ionization (EI) at 70 eV, operating in the full-scan acquisition mode in the m/z range 40-400. The EOs were diluted 1:20 (v/v) with n-hexane before GC-MS analysis.

GC-FID analysis

Chromatographic characterization of EOs was performed on a 7820 gas chromatograph (Agilent Technologies, Milan, Italy) with a flame ionization detector (FID). EOs and the mixture of aliphatic hydrocarbons (C_8–C_{40}) were diluted 1:20 (v/v) with Hex before GC-FID analysis. Helium was used as carrier gas at a flow rate of 1 mL/min with a pressure of 2.5 bar at the column head. The injector and detector temperatures were set at 250 and 300 °C, respectively. EO components were separated on an Agilent Technologies HP-5 crosslinked poly-5% diphenyl–95% dimethyl siloxane (30 m x 0.32 mm i.d., 0.25 mm film thickness) capillary column. The column temperature was initially set at 45 °C, then increased at a rate of 2 °C/min up to 100 °C, then raised to 250 °C at a rate of 5 °C/min and finally maintained for 5 min. The injection volume was 1 μL, with a split ratio 1:20.

Compounds were identified by comparing the retention times of the chromatographic peaks with those of authentic reference standards run under the same conditions and by comparing the linear retention indices (*LRI*s) relative to C_8–C_{40} n-alkanes obtained on the HP-5 column under the above-mentioned conditions with the literature. Peak enrichment by co-injection with authentic reference compounds was also carried out. Comparison of the MS-fragmentation pattern of the target analytes with those of pure components was

performed, by using the National Institute of Standards and Technology (NIST version 2.0d, 2005) mass-spectral database.

The percentage relative amount of individual components was expressed as the percent peak area relative to the total peak area obtained by the GC/FID analysis. Semi-quantitative data were acquired from the mean of two analyses.

RESULTS

Essential oils and hydrosols

After the steam distillation, the EOs were measured, and the yield percentages (w/w) were calculated. The content of EO was 1.87% and 2.74% (w/w) for LA and LI, respectively. Interestingly, the yield of LA was higher than LI's yield. Indeed, LI has been reported to exhibit higher content of EO compared to LA due to the greater number of inflorescences on stems [2–4].

The fresh-distilled EOs were analyzed by GC-FID to quantify the relative percent abundance of the terpenes (Table S1). A total of 32 and 31 compounds were recognized in LA and LI EOs respectively. Interestingly, the chemical composition of the two EOs did not differ as expected according to the European Pharmacopoeia [5], especially for the content of camphor, lavandulyl acetate, 1,8-cineole, borneol, and ocimene. The relative abundances of terpenes in LI EO resulted in agreement with those reported in the literature by several authors [1,6–8], while the composition of LA turned out to be extremely different [2,3]. In particular, high contents of camphor are rarely quantified in this valuable EO, as in the case of Usano-Alemany et *al*. work [9]. This diversity might be to several factors that affect the biosynthetic pathways, such as pedo-climatic conditions, light exposure, cropping technique, and physicochemical soil features [10–13].

Table S1. Semi-quantitative results of the percent chemical composition of LA and LI EOs.The results are the mean of the duplicate analysis performed on the collected EOs.

Compound	*LRI*	*LA*	*LI*
**α-pinene**	930	0.54	0.68
**camphene**	944	0.32	0.36
**sabinene**	970	0.18	0.24
**β-pinene**	973	0.56	0.82
**Oct-1-en-3-ol**	977	0.14	-
**myrcene**	990	1.27	1.52
**3-carene**	1008	0.10	0.18
**α-terpinene**	1013	0.26	0.14

**limonene**	1027	0.65	0.54
**1,8-cineole**	1029	5.27	7.90
**cis-ocimene**	1037	1.39	1.65
**trans-ocimene**	1047	1.27	0.70
**gamma terpinene**	1056	0.10	0.17
**cis linalool oxide**	1071	0.15	0.12
**trans linalool oxide**	1086	0.34	0.33
**linalool**	1105	31.72	27.61
**fenchol**	1113	0.36	0.13
**camphor**	1144	5.68	6.78
**borneol**	1165	2.47	1.86
**lavandulol**	1168	0.40	0.27
**terpinen-4-ol**	1177	1.81	1.08
**α-terpineol**	1190	0.58	0.61
**myrtenal**	1193	0.28	0.19
**carvone**	1246	0.11	0.12
**linalyl acetate**	1265	33.86	35.63
**lavandulyl acetate**	1293	3.47	2.91
**neryl acetate**	1366	0.24	0.29
**α-copaene**	1385	0.49	0.59
**β-caryophyllene**	1423	1.56	1.53
**α-humulene**	1459	1.09	1.25
**ar curcumene**	1486	0.47	0.58
**γ-cadinene**	1519	0.20	0.31
**Total**		97.33	97.07

The hydrosols collected after the steam distillation were extracted in triplicate to isolate the volatile components dissolved in the water. The amount of hydrosol components recovered by solvent extraction represented the 0.101 ± 0.005% and 0.078 ± 0.003 % (w/w) for LA and LI respectively. Both the hydrosols were rich in alcoholic monoterpenes, which represented more than 70% of the whole composition (Table S2). This evidence is in agreement with the higher solubility of the alcohols in aqueous media compared to alkanes, esters, or ketones/aldehydes. Differently from EOs, linalyl acetate represented the 0.1% of the total

composition, accordingly with its instability in water, where the hydrolysis to the alcohol occurs [1]. In addition, several alcohols were detected in the hydrosols but not in the EOs, such as menthol, neomenthol, p-cymen-8-ol, nerol, carvacrol and citronellol. Furthermore, the percentages of borneol, linalool oxides, α-terpineol, and terpinene-4-ol were higher in the hydrosols than in the EOs. These findings might be explained by the fact that their concentrations in the EOs were too low to be detected. On the contrary, due to the limited solubility of several terpenes, their relative abundances resulted higher in the hydrosols. Furthermore, part of them might derive from other monoterpenes after molecular rearrangement due to oxidation and cyclization processes.

Table S2. Semi-quantitative results of the percent chemical composition of LA and LI hydrosols. The results are expressed as mean ± standard deviation of the analysis performed on the three extracts of each hydrosol.

Compound	*LRI*	*LA*	*LI*
**α-pinene**	944	0.26 ± 0.15	0.17 ± 0.01
**camphene**	948	0.26 ± 0.03	0.24 ± 0.02
**β-pinene**	977	0.2 ± 0.02	0.13 ± 0
**1,8 cineole**	1028	2.12 ± 2.06	1.8 ± 1.35
**cis-ocimene**	1037	0.57 ± 0.04	0.49 ± 0.11
**cis linalool oxide**	1070	4.46 ± 0.47	4.62 ± 1.14
**trans linalool oxide**	1087	4.25 ± 0.55	4.13 ± 1
**linalool**	1101	28.67 ± 0.63	32.12 ± 0.47
**camphor**	1143	13.38 ± 0.41	15.63 ± 0.99
**borneol**	1165	10.64 ± 1.31	10.42 ± 1.01
**neomenthol**	1169	1.96 ± 0.26	1.55 ± 0.22
**lavandulol**	1174	1.08 ± 0.06	0.92 ± 0.32
**terpinen-4-ol**	1177	5.58 ± 0.55	3.94 ± 0.14
**menthol**	1182	0.75 ± 0.09	1 ± 0.15
**p-cymen-8-ol**	1185	0.74 ± 0.13	1.1 ± 0.07
**α-terpineol**	1191	11.18 ± 0.28	9.78 ± 0.66
**myrtenal**	1195	0.21 ± 0.01	0.25 ± 0.02
**verbenone**	1208	0.44 ± 0	0.55 ± 0.04
**nerol**	1224	0.33 ± 0.03	0.31 ± 0.03
**citronellol**	1233	4.53 ± 0.04	2.48 ± 0.33
**carvone**	1243	0.18 ± 0.04	0.19 ± 0.01
**neral**	1255	0.15 ± 0	0.17 ± 0.02
**piperitone**	1259	1.94 ± 0.07	1.65 ± 0.27

**linalyl acetate**	1262	0.13 ± 0.02	0.1 ± 0
**geranial**	1276	0.34 ± 0	0.39 ± 0.11
**lavandulyl acetate**	1295	0.13 ± 0	0.1 ± 0
**carvacrol**	1303	0.66 ± 0.09	0.38 ± 0.01
**Total**		95.16 ± 1.28	94.49 ± 0.43

TPC and TFC content

The concentrations of total polyphenols and flavonoids were determined by using a standard curve prepared with gallic acid and quercetin solutions. The calibration curves are reported in Figure S1. The equations obtained from the linear regression were Y = 0.9755X - 0.02728 and Y = 1.600X + 0.01067 for gallic acid and quercetin respectively.

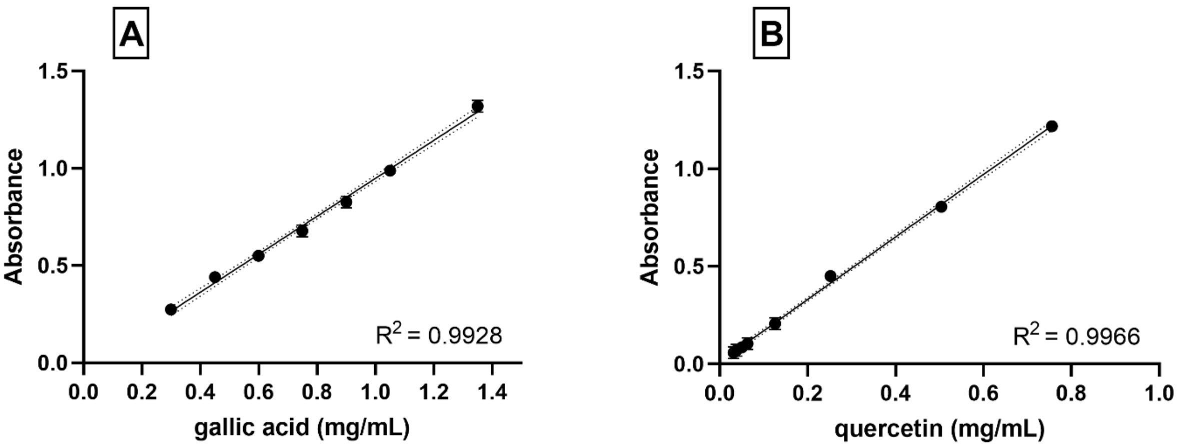

Figure S1. Calibration curves of gallic acid (A) and quercetin (B) employed for determining the content of polyphenols and flavonoids in the extracts.

REFERENCES

1. Truzzi, E.; Benvenuti, S.; Bertelli, D.; Francia, E.; Ronga, D. Effects of Biostimulants on the Chemical Composition of Essential Oil and Hydrosol of Lavandin (Lavandula x intermedia Emeric ex Loisel .) Cultivated in Tuscan-Emilian Apennines. *Molecules* **2021**, *26*, 6157, doi:10.3390/molecules26206157.

2. Renaud, E.N.C.; Charles, D.J.; Simon, J.E. Composition from 10 Cultivars of Organically Grown Lavender and Lavandin. *J. Essent. Oil Res. 13*, 269–273, doi:10.1080/10412905.2001.9699691.

3. Lafhal, S.; Vanloot, P.; Bombarda, I.; Kister, J.; Dupuy, N. Identification of metabolomic markers of lavender and lavandin essential oils using mid-infrared spectroscopy., doi:10.1016/j.vibspec.2016.04.004ï.

4. Jianu, C.; Pop, G.; Gruia, A.T.; Horhat, F.G. Lavandula angustifolia) and lavandin (Lavandula x intermedia) grown in Western Romania. *Int. J. Agric. Biol.* **2013**, *15*, 772–776.

5. *European Pharmacopoeia*; Council of Europe, Ed.; 10th ed.; Strasbourg, 2021;

6. Carrasco, A.; Martinez-Gutierrez, R.; Tomas, V.; Tudela, J. Lavandin (Lavandula × intermedia Emeric ex Loiseleur) essential oil from Spain: Determination of aromatic profile by gas chromatography-mass spectrometry, antioxidant and lipoxygenase inhibitory bioactivities. *Nat. Prod. Res.* **2016**, *30*, 1123–1130, doi:10.1080/14786419.2015.1043632.

7. Bombarda, I.; Dupuy, N.; Da, J.P.L. Van; Gaydou, E.M. Comparative chemometric analyses of geographic origins and compositions of lavandin var. Grosso essential oils by mid infrared spectroscopy and gas chromatography. *Anal. Chim. Acta* **2008**, *613*, 31–39, doi:10.1016/j.aca.2008.02.038.

8. Barocelli, E.; Calcina, F.; Chiavarini, M.; Impicciatore, M.; Bruni, R.; Bianchi, A.; Ballabeni, V. Antinociceptive and gastroprotective effects of inhaled and orally administered Lavandula hybrida Reverchon "grosso" essential oil. *Life Sci.* **2004**, *76*, 213–223, doi:10.1016/j.lfs.2004.08.008.

9. Usano-Alemany, J.; Herraiz Peñalver, D.; Cuadrado Ortiz, J.; De López, B.B.; Ruiz, O.S.; Palá-Paúl, J. Ecological production of lavenders in Cuenca province (Spain). A study of yield production and quality of the essential oils. *Bot. Complut.* **2011**, *35*, 147–152.

10. Hassiotis, C.N.; Ntana, F.; Lazari, D.M.; Poulios, S.; Vlachonasios, K.E. Environmental and developmental factors affect essential oil production and quality of Lavandula angustifolia during flowering period. *Ind. Crops Prod.* **2014**, *62*, 359–366, doi:10.1016/j.indcrop.2014.08.048.

11. Fernández-Sestelo, M.; Carrillo, J.M. Environmental effects on yield and composition of essential oil in wild populations of spike lavender (Lavandula latifolia medik.). *Agric.* **2020**, *10*, 1–18, doi:10.3390/agriculture10120626.

12. Moghaddam, M.; Mehdizadeh, L. Chemistry of Essential Oils and Factors Influencing Their Constituents. In *Soft Chemistry and Food Fermentation*; Elsevier, 2017; pp. 379–419.

13. Arabaci, O.; Bayram, E.; Baydar, H.; Ferhan Savran, A.; Karadogan, T.; Ozay, N. *Chemical Composition, Yield and Contents of Essential Oil of Lavandula hybrida Reverchon Grown under Different Nitrogen Fertilizer, Plant Density and Location*; 2007; Vol. 19;.
